# Novel Imaging Finding and Novel Mutation in an Infant with Molybdenum Cofactor Deficiency, a Mimicker of Hypoxic-Ischaemic Encephalopathy

**Published:** 2018

**Authors:** Sangeetha YOGANATHAN, SniyaVALSA SUDHAKAR, Maya THOMAS, Atanu KUMAR DUTTA, Sumita DANDA, Mahalakshmi CHANDRAN

**Affiliations:** 1 Department of Pediatrics Neurology Sciences, Christian Medical College, Tamil Nadu, India; 2 Department of Pediatrics Radiology, Christian Medical College, Tamil Nadu, India; 3 Department of Medical Genetics, Christian Medical College, Tamil Nadu, India

**Keywords:** Seizure, Encephalomalacia, Sulfite, Molybdenum cofactor

## Abstract

Molybdenum cofactor deficiency is a rare metabolic disorder manifesting with early onset seizures, developmental delay, microcephaly, and spasticity. In this report, we describe a three-month-old infant with neonatal onset, poorly controlled seizures, developmental delay, microcephaly, spastic quadriparesis and visual insufficiency. Magnetic resonance imaging of brain had shown cystic encephalomalacia involving bilateral parieto-occipital lobe and elevated lactate in magnetic resonance spectroscopy. Restricted diffusion noted along the corticospinal tract in our case is a novel imaging finding in patients with molybdenum cofactor deficiency. Low serum uric acid and elevated urine sulfite excretion were observed. A novel homozygous mutation was detected in exon 4 of molybdenum cofactor synthesis 2 (*MOCS2*) gene. Early infantile or neonatal onset seizures, developmental delay, microcephaly and cystic encephalomalacia in neuroimaging mimicking hypoxic-ischaemic encephalopathy should raise the suspect for molybdenum cofactor deficiency. Screening of all neonates for urinary sulfite metabolites would help in early diagnosis and management. Early diagnosis and treatment with cyclic pyranopterin monophosphate could arrest the progression of molybdenum cofactor deficiency type A. More research is needed to explore further treatment options in this otherwise lethal disorder.

## Introduction

Molybdenum cofactor deficiency (MoCD) is a rare metabolic cause of neonatal onset neurological symptoms, developmental delay, seizures and motor abnormalities, first described in 1978 (1). If undiagnosed, this disorder can result in rapid progressive encephalopathy and death. Early diagnosis is important in this lethal disorder as treatment with cyclic pyranopterin monophosphate (cPMP) could arrest the progression of MoCD type A (2). 

We describe an infant with MoCD having early onset refractory seizures and imaging findings mimicked hypoxic-ischemic encephalopathy. Restricted diffusion was noted along the corticospinal tract in our patient. It is a novel imaging finding in patients with MoCD and a novel mutation was also detected in the exon 4 of molybdenum cofactor synthesis 2 (*MOCS2*) gene.

## Case report

A three-month-old boy born first in birth order to a non-consanguineously married Indian couple was referred for the evaluation of global developmental delay and refractory seizures. Informed consent was taken from the mother. Mother had no antenatal risk factors. The baby was delivered full term by normal vaginal delivery with a birth weight of 3200 g. Neonatal transition was uneventful. Jaundice was noticed on third day of life and the maximum bilirubin recorded was 16.3 mg/dL. There was no ABO or Rh incompatibility and he was discharged home on exclusive breastfeeds. He was readmitted on eighth day of life with history of poor feeding and lethargy. Baby was initiated on intravenous fluids and antibiotics, after sending samples for sepsis screen. Sepsis workup was negative and he was discharged home, as there was a clinical improvement. At the end of second week of life, he presented to the Paediatric Emergency Ward with frequent episodes of multifocal clonic seizures. Blood sugar on arrival was 77 mg/dL and the seizures were managed with loading dose of phenobarbitone and phenytoin. There were no skin rashes, abnormal urine odor, fast breathing or recurrent hiccups. From second month of age, child had developed multiple episodes of myoclonus per day and seizures were poorly controlled despite treatment with three antiepileptic medications in adequate doses.

There were no neurocutaneous markers or dysmorphism. Occipto-frontal circumference was 36.5 cm. Ocular examination did not reveal any ectopia lentis. He was awake and irritable with inconsolable cry. He was also observed to have poor quality and quantity of spontaneous limb movements. He did not fix or track the light and fundus examination was normal. Pupillary reflexes were normal. Profound axial hypotonia with appendicular hypertonia and brisk deep tendon reflexes were present. There were no involuntary movements. Anterior fontanelle was normal.

Complete blood count, blood urea, creatinine, electrolytes and liver function test were normal. Serum uric acid was less than 0.5 mg/dL. Blood lactate, ammonia, and serum amino acids were normal. Urinary organic acids were not detected. Cerebrospinal fluid analysis was normal. Electroencephalography done in the first month of life was normal. Freshly voided urine analysis by calorimetric method had revealed an increased sulfite excretion (patient value-1425 µmoles/L; normal- 0-96.7 µmoles/L). 

**Fig 1:**
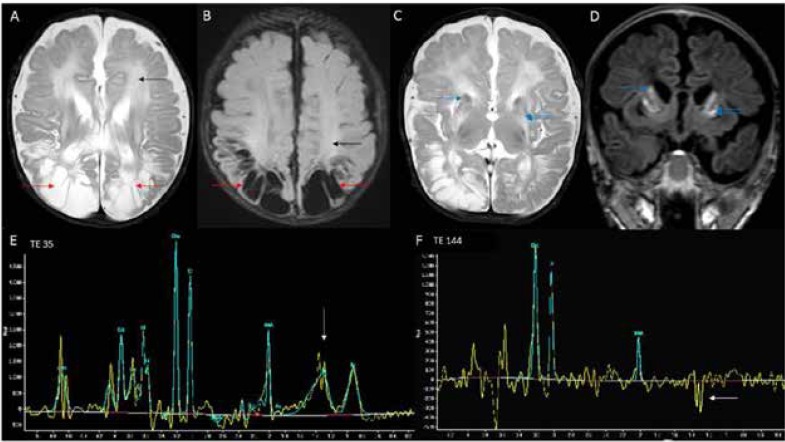
MRI brain T2 axial (A) and FLAIR (B) images show bilateral parieto-occipital cystic encephalomalacia (red arrows) and deep white matter hyperintensities (black arrows). Volume loss and signal changes of caudate (blue arrow- single) and putamen (blue arrows- double) are seen in T2 axial (C) image. Corresponding T1 coronal image (D) shows hyperintense signal (blue arrows- single and double) which could represent early calcification. Also note the sparing of thalamus. MRS from parenchyma shows peak at TE 35 (E) with inversion at TE 144 (F) suggestive of lactate

**Fig 2:**
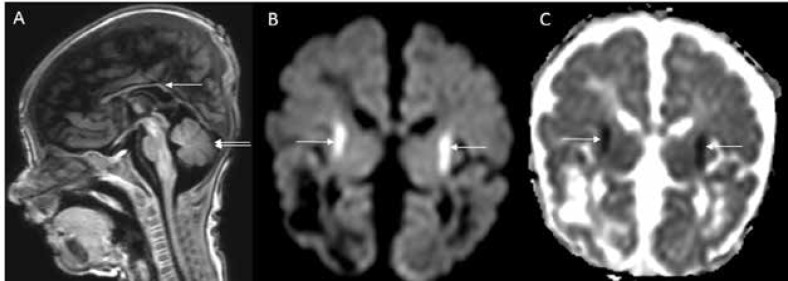
MRI brain T1 sagittal image (A) shows diffuse thinning of corpus callosum (white arrow); brainstem appear normal and cerebellum also appear normal (double arrows). DWI (B) and ADC (C) images show restricted diffusion along corticospinal tracts at the corona radiata (white arrows

Brain magnetic resonance imaging (MRI) (Figure 1A-C) revealed extensive cystic encephalomalacia involving bilateral parieto-occipital lobe and signal changes involving caudate, putamen and deep white matter. Basal ganglia showed focal T2 hypointensity and corresponding T1 hyperintensity (Figure 1D) predominantly involving head of the caudate and putamen bilaterally. This is most suggestive of early calcification although confirmatory CT brain was not done. Diffuse callosal thinning was also noted (Figure 2A). Restriction of diffusion was seen along the corticospinal tract in the diffusion weighted and apparent diffusion coefficient images (Figure 2B-C). Magnetic resonance spectroscopy (MRS) had shown an elevated lactate peak (Figure 1E-F). 

Diagnosis of MoCD was suspected based on the clinical, radiological findings and biochemical markers. *MOCS1 *and *MOCS2* genes were sequenced by Sanger sequencing (primer sequences and conditions are available on request). A homozygous mutation was detected in exon 4 of *MOCS2* gene: NM_004531.4: c.218T>C which leads to substitution of Leucine at position 73 to Proline. Leucine at position 73 is a highly conserved residue across different species. This is a novel mutation which is not listed in dbsnp, ExAc and HGMD databases and predicted to be pathogenic by MutationTaster, FATHMM-MKL, LRT, SIFT, PROVEAN, and POLYPHEN prediction (DANN score 0.9984).

Child had partial head control but there was no midline hand regard, social smile, visual fixation to toys or startle response to loud sounds during a follow-up neurodevelopmental assessment at five months of age. He had poorly controlled seizures, microcephaly, spastic quadriparesis and cerebral visual impairment. Child was managed with anticonvulsants and infant stimulation therapy. Despite all supportive measures and optimization of antiepileptic medications, child succumbed at nine months of age.

## Discussion

Rare presentation of late-onset movement disorder has also been reported (3). MOCD results in the loss of function of sulfite oxidase, aldehyde oxidase, xanthine dehydrogenase and mitochondrial amidoxime reducing component (4). MOCD leads to an increased urinary excretion of sulfite, thiosulfate, S- sulfocysteine, taurine, and hypotaurines. MOCD leads to a decrease in the conversion of xanthine to uric acid resulting in low serum uric acid level. Sulfite accumulation is toxic to the proteins and sulfur containing aminoacids, thereby resulting in deranged cellular metabolism (4). Elevated sulfite has a neurotoxic effect and results in neuronal death (4). 

Early onset refractory seizures, microcephaly, encephalopathy with or without dislocation of lens should cast a clue for the diagnosis of MOCD or isolated sulfite oxidase deficiency. Screening tests for the diagnosis of MOCD are low blood uric acid and elevated excretion of sulfite, hypoxanthine, and xanthine in urine. Our patient had classical clinical phenotype, low blood uric acid level and elevated urinary excretion of sulfite. However, absence of elevated urine sulfite excretion may also occur in patients with MOCD due to rapid degradation resulting from delay in processing of the sample and therefore, it is essential to test sulfite levels in a freshly voided urine sample (5).

Brain MRI findings in MOCD closely resemble the pattern observed in neonates with hypoxic-ischaemic encephalopathy. MRI findings described in patients with isolated sulfite oxidase or MOCD are cerebral edema, cerebral atrophy, cerebellar atrophy, calcification in basal ganglia, cystic encephalomalacia, ventriculomegaly, signal changes in internal capsule, external capsule and subcortical white matter tracts, delayed myelination, cystic changes in basal ganglia, hypoplasia of corpus callosum, brainstem and basal ganglia (6,7). MRI findings observed in our patient are cystic encephalomalacia, signal changes in the white matter and basal ganglia, thinning of corpus callosum. Diffusion restriction noted along the corticospinal tract in our case is a novel imaging finding. Widespread or non-uniform pattern of diffusion restriction in the cortical and subcortical regions have been reported in infants with MOCD deficiency (8,9). Elevated lactate peak was observed in our patient as described in a previous report (2). It could possibly be due to energy failure resulting from neurotoxic effect of sulfite on the mitochondria.

Three types of MOCD have been described based on the genetic defect that includes type 1 due to *MOCS1* mutation, type 2 due to *MOCS2 *mutation and type 3 due to *GEPHYRIN* mutation. A novel homozygous mutation was identified in exon 4 of *MoCS2* gene in our patient and has not been reported previously in the literature.

Patients with MOCD type A have deficiency of CPMP and early initiation of treatment with 80-320 mcg/kg/d of CPMP was found to be beneficial (2, 10). Other measures tried in the management of children with isolated sulfite oxidase or molybdenum cofactor deficiency includes administration of anticonvulsants, restriction of sulfur-containing amino acids in diet, use of dextromethorphan, use of d-penicillamine to bind sulfites and addressing the feeding and respiratory difficulties (11-14).

**In conclusion, **MOCD must be considered in all neonates and infants with microcephaly, refractory seizures, and developmental delay with or without ectopia lentis. Infants with MOCD have distinctive imaging findings mimicking hypoxic-ischaemic encephalopathy. Screening of all neonates for urinary sulfite metabolites helps in early diagnosis. More research is needed to explore further treatment options in this otherwise lethal disorder.
